# Distribution of cognitive domains and neuropathology of cases with mild cognitive impairment

**DOI:** 10.1002/alz.092655

**Published:** 2025-01-03

**Authors:** Andrew Ho, Parichita Choudhury, Charles Adler, David Shprecher, Shyamal Mehta, Holly Shill, Erika Driver‐Dunckley, Christine Belden, Geidy E Serrano, Thomas G. Beach, Alireza Atri, Cécilia Tremblay

**Affiliations:** ^1^ Banner Sun Health Research Institute, Sun City, AZ USA; ^2^ Mayo Clinic, Scottsdale, AZ USA; ^3^ Mayo Clinic Arizona, Scottsdale, AZ USA; ^4^ Barrow Neurological Institute, Phoenix, AZ USA; ^5^ Civin Laboratory for Neuropathology, Banner Sun Health Research Institute, 10515 W Santa Fe Drive, Sun City, AZ 85351, Sun City, AZ USA; ^6^ Banner Sun Health Research Institute, Phoenix, AZ USA

## Abstract

**Background:**

Mild Cognitive Impairment (MCI) is a pre‐dementia state where impaired cognitive domains (memory, executive, visuospatial processing, language) may predict underlying pathology, e.g. Alzheimer’s disease(AD), Lewy body (LB), and other. Delineating neuropathological heterogeneity in MCI and their associations with neuropsychological profiles are important in identifying individuals at risk of further deterioration. We aimed to characterize the neuropathological heterogeneity in cases with a diagnosis of MCI.

**Method:**

Cases with a final consensus cognitive diagnosis of MCI (n = 128) prior to death were selected from the Arizona Study of Aging and Neurodegenerative disorders (AZSAND). Groups were divided into amnestic (aMCI) and non‐amnestic MCI (naMCI), single and multi‐domain impairment, and specific domain impairment (executive, language, visuospatial) was explored. Presence and severity of neuropathology including AD, LB, cerebral white matter rarefaction (CWMR), cerebral amyloid angiopathy (CAA), cerebral infarcts and microinfarcts (cortical and subcortical), TAR DNA‐binding protein 43 (TDP‐43), and apolipoprotein E (ApoE) status were compared between MCI subtypes.

**Result:**

The number of cases meeting neuropathological criteria for AD was not different between aMCI (36.4%) and naMCI (22.6%) while co‐existent PD was less frequent in aMCI (19.5% vs 38.7%). When compared to naMCI (n = 31), the aMCI (n = 85) group had an older age of death (p = 0.001), less educational years (p = 0.04), a higher number of subcortical infarcts (p = 0.012), a higher regional density of neurofibrillary tangles (p = 0.027) and a lower density of LB (p = 0.015) in the temporal lobe, and CWMR in tended to be more severe in the frontal lobe (p = 0.052). Those with single domain aMCI had less CWMR (p = 0.001) compared to the multidomain. Within the naMCI group, those with single domain MCI had higher proportions of CAA (p = 0.042), and lower LB density (p = 0.019) compared to multidomain. CWMR was more severe (p = 0.026) in

**Conclusion:**

A high heterogeneity was observed between MCI subtypes. While AD pathology was similar between groups, regional anatomical differences were observed. Vascular pathology including subcortical infarcts, CWMR and CAA appear to be commonly associated with both aMCI and naMCI respectively while CWMR was associated with executive dysfunctions. CWMR, CAA and LBs appear to be more frequent in multidomain aMCI and naMCI respectively.

